# Diffuse nesidioblastosis with hypoglycemia mimicking an insulinoma: a case report

**DOI:** 10.1186/1752-1947-6-332

**Published:** 2012-10-02

**Authors:** Chiara Ferrario, Delphine Stoll, Ariane Boubaker, Maurice Matter, Pu Yan, Jardena J Puder

**Affiliations:** 1 Department of Endocrinology, University Hospital of Lausanne (CHUV), Rue du Bugnon 44, 1011, Lausanne, Switzerland; 2 Department of Nuclear medicine, University Hospital of Lausanne (CHUV), Rue du Bugnon 44, 1011, Lausanne, Switzerland; 3 Department of Surgery, University Hospital of Lausanne (CHUV), Rue du Bugnon 44, 1011, Lausanne, Switzerland; 4 Department of Pathology, University Hospital of Lausanne (CHUV), Rue du Bugnon 25, 1011, Lausanne, Switzerland

**Keywords:** In-pentetreotide scintigraphy, Adult, Hypoglycemia, Imaging, Insulinoma, Nesidioblastosis

## Abstract

**Introduction:**

We describe a case of diffuse nesidioblastosis in an adult patient who presented with exclusively fasting symptoms and a focal pancreatic ^111^In-pentetreotide uptake mimicking an insulinoma.

**Case presentation:**

A 23-year-old Caucasian man had severe daily fasting hypoglycemia with glucose levels below 2mmol/L. Besides rare neuroglycopenic symptoms (confusion, sleepiness), he was largely asymptomatic. His investigations revealed low venous plasma glucose levels, high insulin and C-peptide levels and a 72-hour fast test that were all highly suggestive for an insulinoma. Abdominal computed tomography and magnetic resonance imaging did not reveal any lesions. The sole imagery that was compatible with an insulinoma was a ^111^In-somatostatin receptor scintigraphy that showed a faint but definite focal tracer between the head and the body of the pancreas. However, this lesion could not be confirmed by endoscopic ultrasonography of the pancreas. Following duodenopancreatectomy, the histological findings were consistent with diffuse nesidioblastosis. Postoperatively, the patient continued to present with fasting hypoglycemia and was successfully treated with diazoxide.

**Conclusion:**

In the absence of gastrointestinal surgery, nesidioblastosis is very rare in adults. In addition, nesidioblastosis is usually characterized by post-prandial hypoglycemia, whereas this patient presented with fasting hypoglycemia. This case also illustrates the risk for a false positive result of ^111^In-pentetreotide scintigraphy in the case of nesidioblastosis. Selective arterial calcium stimulation and venous sampling is the most reliable procedure for the positive diagnosis of insulinoma or nesidioblastosis and should be used to confirm any suspicion based on imaging modalities.

## Introduction

Whereas nesidioblastosis is the primary cause of persistent hyperinsulinemic hypoglycemia in infants, noninsulinoma pancreatogenous hypoglycemia syndrome (NIPHS) is very infrequent in adults [1-3]. Nesidioblastosis is the histological equivalent of noninsulinoma pancreatogenous hypoglycemia syndrome and refers to an increase in the size and number of pancreatic beta cells islets with focal or diffuse hypertrophy and hyperfunction [4]. Nesidioblastosis was first described by Laidlaw in 1938 [5]. The first adult case was reported in 1975 [6]. Since then, fewer than 100 cases have been reported, all of which occurred in the absence of gastrointestinal surgery [7-12].

The confirmative diagnosis of NIPHS remains very challenging despite the improvement of both morphological and functional imaging techniques [13]. In most cases, the diagnosis is made postoperatively and is based on histological findings [14].

Clinically, it is often difficult to distinguish NIPHS from insulinoma although some authors suggest that post-prandial hypoglycemia could be indicative for NIPHS. NIPHS is the cause of endogenous hyperinsulinemia in less than 5% of the cases [2,3] making this diagnosis unlikely in patients presenting with hypoglycemic symptoms.

We describe an unusual case of nesidioblastosis with fasting hypoglycemia and ^111^In-pentetreotide focal uptake in the pancreas, mimicking an insulinoma.

## Case presentation

A hitherto healthy 23-year-old Caucasian male student reported a history of several intermittent episodes of absence for over a year. According to his family, such episodes recurred every 2 months. During the episodes, he was conscious, but felt sleepy, his vision was blurred and he was unable to talk. In addition, he suffered one episode of concomitant jaw paralysis. These episodes happened most frequently upon awakening, but sometimes also late in the afternoon. The family had noticed that his symptoms improved continuously within 30 minutes of having eaten honey, sugar or fruits. Every episode was usually followed by headaches. He also mentioned a weight gain of 6kg over the past year. His physical examination was normal and a magnetic resonance imaging (MRI) and electroencephalogram with sleep deprivation failed to demonstrate any abnormality. However, never was the patient evaluated while symptomatic. He was referred to our endocrinology out-patient clinic to exclude a metabolic disorder. He had no family history of any metabolic or endocrine disorders.

In the meantime, his general practitioner taught the patient to self-monitor his capillary blood glucose levels. While completely asymptomatic, his fasting glucose levels ranged between 2.3mmol/L and 3.0mmol/L. None of the blood glucose measures was done during an episode of absence.

Laboratory findings on two different occasions revealed low venous fasting plasma glucose concentrations (2.6mmol/L and 1.6mmol/L) with high insulin (15.5mU/L and 55.3mU/L) and C-peptide (0.762nmol/L and 0.53nmol/L) levels. A 72-hour fasting test had to be stopped after 18 hours because of a glucose level of 1.8mmol/L and a mini-mental state score (MMSE) of 24 out of 30. After an intravenous injection of 1mg of glucagon, the glucose level increased to 4.7mmol/L and the MMSE to 29 out of 30. A plasma sulfonylureas screen test at the beginning and at the end of the fasting study was negative. Anti-insulin antibodies were negative. Insulin and C-peptide levels increased during the fasting test. These results confirmed our clinical suspicion of an insulinoma.

**Figure 1:**
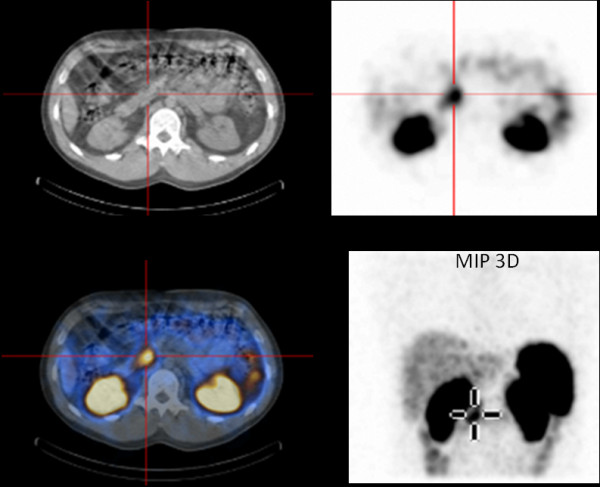
**Somatostatin receptor scintigraphy (OctreoScan®) of the patient: single-photon emission computed tomography was performed 3 hours after injection of 200MBq**^**111**^**In-pentetreotide and showed a focal uptake at the junction between the head and the body of the pancreas.** MIP 3D; Maximum intensity projection, three dimensional.

**Figure 2:**
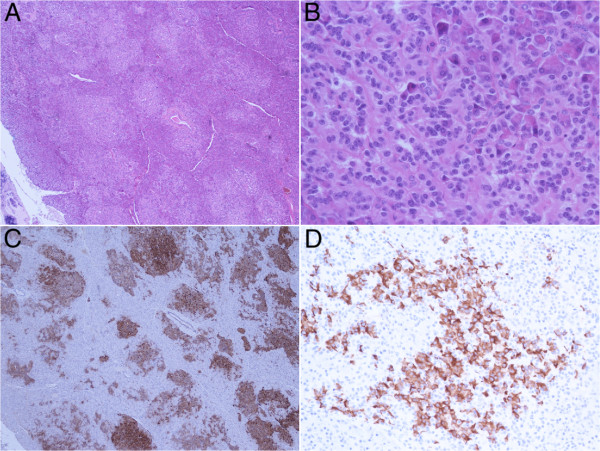
Enlarged pancreatic islet with irregular outline (×4).

**Figure 3:**
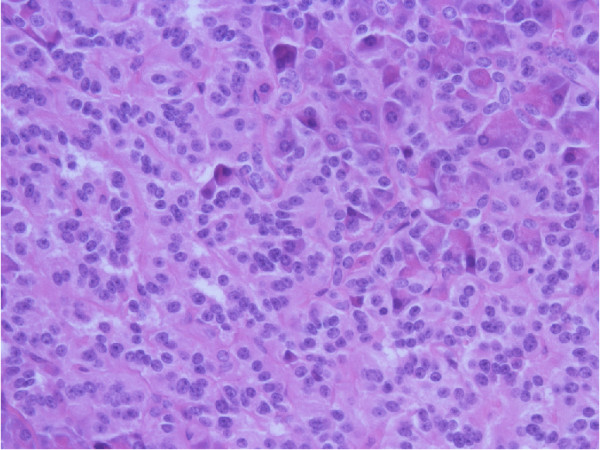
Pancreatic beta cells showing increased nuclear size and prominence of nucleoli (×40).

**Figure 4:**
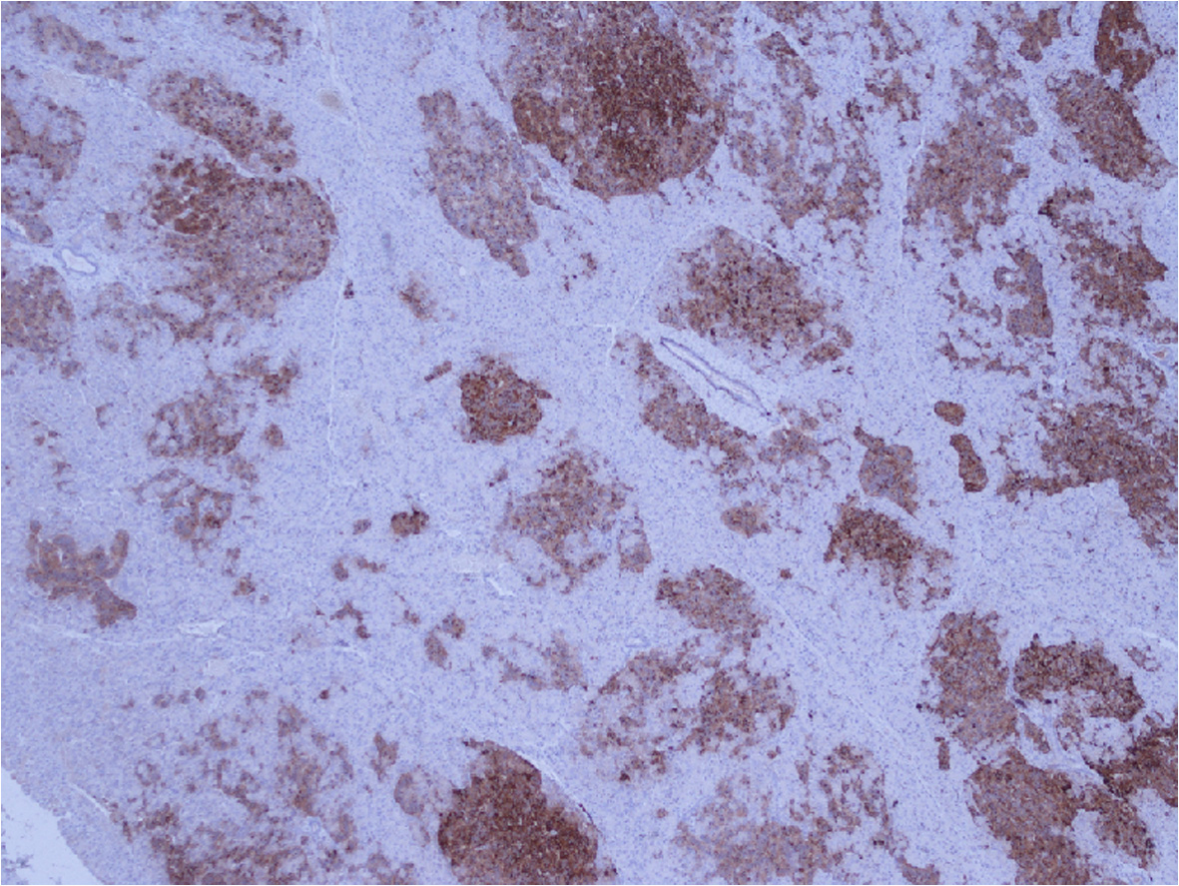
Immunostaining of chromogranin and synaptophysin highlighted the enlarged and irregular islets (×10).

**Figure 5:**
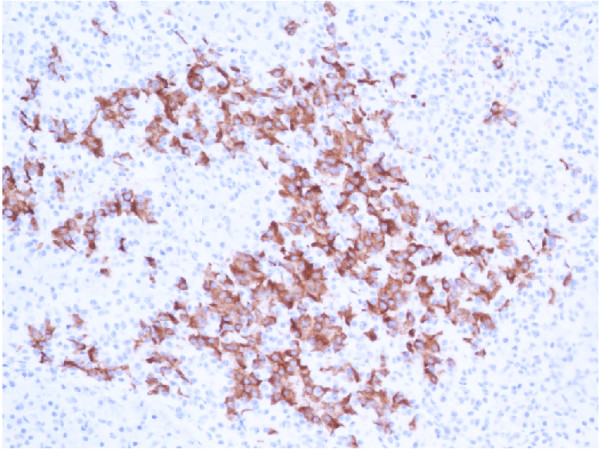
Approximately half of all cells within the pancreatic islets were stained with insulin (×20).

An abdominal computed tomography (CT) and MRI did not reveal any lesions. A subsequent somatostatin receptor scintigraphy (SRS) performed 4 hours and 24 hours after injection of 190MBq ^111^In-pentetreotide (OctreoScan®) showed a faint but definite, albeit discrete, focal abnormality at the junction between the head and body of the pancreas, compatible with an insulinoma (Figure 1). Nevertheless, endoscopic ultrasound of the pancreas failed to confirm this finding. We decided not to perform selective intra-arterial calcium stimulation with hepatic venous sampling at this stage because we relied on the results of the SRS. We initially planned to complete the investigations by performing a glucagon-like peptide-1 (GLP-1) receptor scintigraphy to confirm the lesion found only on SRS. The patient, nonetheless, refused this supplementary imaging procedure. After a multidisciplinary discussion, a ^111^In-pentetreotide radio-guided surgery was performed. The day before surgery, the patient received 200MBq of ^111^In-pentetreotide and single-photon emission computed tomography (SPECT) CT, which confirmed the focal uptake located between the head and the body of the pancreas described above. During surgery, the nuclear gamma probe detected the increased uptake that was suspected to be the lesion within the head and the body of the pancreas, but intraoperative ultrasound failed to detect any morphological abnormality. Biopsies were undertaken in the area of this maximal signal intensity detected by the gamma probe. However, the frozen sections were not conclusive for an insulinoma. Metallic clips were put around the biopsy site and the abdomen was closed. Another SRS was performed 2 weeks after this first intervention and confirmed that the uptake was located between the surgical clips, exactly at the same location as it had been seen on the first SPECTCT. As the tumor was located deeper in the head of the pancreas, a pylorus preserving duodenopancreatectomy was performed after having obtained the patient’s consent. Macroscopic examination of the resection specimen identified no tumor. Microscopically, the lobular architecture of the exocrine pancreas was preserved and no ductuloinsular complexes were observed in any specimens. Some of the islets were enlarged and varied in size; some measuring 0.2cm and randomly scattered throughout the pancreatic parenchyma. The outlines of the islets were more irregular than normal (Figure 2). A consistent finding in all specimens was the presence of multiple enlarged islet cells showing increased nuclear size with prominent nucleoli and clear cytoplasm (Figure 3). There was no mitotic activity. The enlarged and irregular islets were highlighted with immunohistochemical staining by using antibodies against chromogranin and synaptophysin (Figure 4). Approximately half of all cells within the pancreatic islets were stained with insulin (Figure 5). By contrast, glucagon and somatostatin cells did not have any cytologic abnormalities. Insulin-positive cells were also scattered throughout the parenchyma. These features, combined with the clinical and radiological findings, are consistent with diffuse nesidioblastosis.

Noting that multiple endocrine neoplasia mutations were described in conjunction with nesidioblastosis, a menin gene mutation with polymerase chain reaction enzymatic amplification was performed and was found to be negative. Calcium, prolactin and insulin-like growth factor 1 levels were within normal values.

Postoperative recovery was complicated by a wound infection. Following surgery, the hypoglycemic episodes continued and a diffuse nesidioblastosis of the pancreatic distal body and tail was suspected. The patient’s diet was adapted to provide regular intake of carbohydrates, especially early in the morning, but the patient’s symptoms persisted. To avoid further pancreatic resection in such a young patient, a verapamil treatment was initiated (40mg twice daily, increasing up to 80mg three times daily, over 1 week). The patient, however, experienced severe headaches and palpitation after 3 weeks and stopped the medication. Diazoxide was then introduced; initially 100mg twice daily, increasing to 100mg three times a day, which resulted in a normalization of his glucose level with peaks of up to 7.2mmol/L. A 72-hour subcutaneous glucose monitoring testing, undertaken through a glucosensor (CGMS Sensor®), revealed normal glycaemia levels.

Four months after surgery, the patient experienced abdominal pain, more frequent stools (three times a day) and weight loss (1kg/week). Stool examination showed high fat levels of 89g per 24 hours (normal value 5g to 7g per 24 hours). Therefore, an enzyme substitution was initiated and dietary advice provided in order to treat the postoperative malabsorption.

## Discussion

Based on the clinical presentation and the biological findings, we were quite confident that the patient had an insulinoma localized either in the head of the pancreas or in the duodenum. The endogenous hyperinsulinemic hypoglycemia was confirmed and one localization procedure, the SRS, was positive. Interestingly, a positive SRS has been described in three other reports of nesidioblastosis as a localized focal uptake [3,11]. SRS is known to have a low sensitivity for insulinoma, being positive in only 50% of cases, and false positive results of SRS in NIPHS have been reported [13]. Nesidioblastosis is a challenge and diagnosis is rarely made before surgery [15]. The histopathological diagnosis of nesidioblastosis has primarily been histological, as in this case, but the criteria employed to make the diagnosis in adults have, until recently, been somewhat unclear. Recently proposed criteria are: (1) exclusion of an insulinoma by macroscopic, microscopic and immunohistochemical examination; (2) multiple beta cells with an enlarged and hyperchromatic nucleus and abundant clear cytoplasm; (3) islets with normal spatial distribution of the various cell types; and (4) no proliferative activity of endocrine cells [15].

Retrospectively, the scintigraphic suspicion should have been further confirmed before surgery. Selective arterial calcium stimulation and venous sampling is a useful tool for the diagnosis and treatment decision of NIPHS, especially with respect to the extent of pancreatectomy. The sensitivity of this technique is reported to be nearly 100% for the diagnosis of insulinomas and should be able to discriminate the focal insulinoma from the more diffuse pattern of nesidioblastosis [10,13,15]. It is possible that the selective arterial calcium stimulation would have shown increased insulin levels following stimulation from the gastroduodenal artery and in a lower proportion from the superior mesenteric and from the splenic arteries as well. New diagnostic options such as dihydroxyphenylalanine (DOPA)-positron emission tomography and, the more recent, GLP-1 scintigraphy, could have been useful to confirm the localization of the suspected insulinoma. However, their value in discriminating the focal insulinoma from the diffuse nesidioblastosis has not yet been demonstrated. Before proceeding to the surgery, we proposed to the patient to conduct additional diagnostic examinations to confirm the scintigraphic findings, but he refused for personal reasons. In any case, the outside nuclear department performing the GLP-1 scintigraphy felt that this test would not provide any additional information.

Persistent hyperinsulinemic hypoglycemia of infancy (PHHI) can occur as a result of mutations in the subunits that form the adenosine-5'-triphosphate (ATP)-sensitive potassium channel (K+ATP) in pancreatic beta cells, which play a major role in modulating insulin secretion from the beta cells. Mutations have been shown in the genes for these subunits, namely for the plasma membrane sulfonylurea receptor (SUR1), ABCC8, and its associated inwardly rectifying potassium channel (KIR6.2) KCNJ11. These genetic defects are known to be relevant in the development of PHHI and at least some of them are very likely to be prevalent also in adults with the disease. Although the mode of inheritance is autosomal recessive in some and dominant in others, spontaneous mutations may of course be of importance too. We analyzed the family history of the patient and found no specific features in other family members (e.g. gestational diabetes in females, unexplained diabetes in non-obese individuals etc.). The favorable response to diazoxide may even give a hint towards which mechanisms may be involved. Specifically, diazoxide which acts on K+ATP channels seems to need intact ABCC8 to be able to show its effects. Because many of the potential mutations in the genes responsible are well known, we could have performed genetic analysis in order to clarify the situation, especially because the patient was young and could have children in the future. To this date, the patient did not wish to perform any other test, but a possible genetic analysis should be discussed with him in the future.

In cases of nesidioblastosis, the extent of surgery is controversial. Ideally, surgery should not be performed without a preoperative localization technique. If laparoscopy or laparotomy cannot detect an insulinoma, then Rostambeigi and Thompson recommend that surgery should stop so as to avoid performing a blind distal pancreatic resection, and investigations should be completed by selective arterial stimulation and venous sampling [16]. Recommendations are based on small series, case reports and experience in children with nesidioblastosis. The preferred option is an extended left-sided pancreatic resection or a Whipple’s procedure with 70% to 90% pancreas resection [10,15], followed by partial resection (<60%) and subtotal to total pancreatectomy (>90%) [13]. Conservative therapy is sometimes suggested. Diazoxide, octreotide and verapamil could be effective and safe alternative conservative therapies when surgery has failed or is considered to be too risky [8,10]. Patients’ information should include the necessity for multiple diagnostic procedures and the postoperative risks for pancreatic exocrine insufficiency and diabetes. In the present case, total pancreatectomy was not proposed due to the young age and the consecutive morbidity of such a procedure [7,13].

## Conclusion

Nesidioblastosis is very rare in adults in the absence of previous gastrointestinal surgery. This case illustrates the difficulties and limitations of diagnostic imaging procedures and the risk for a false positive result of ^111^In-pentetreotide scintigraphy in case of nesidioblastosis. Selective arterial calcium stimulation and venous sampling is the most reliable procedure for the positive diagnosis of insulinoma or nesidioblastosis and should be used to confirm any suspicion based on imaging modalities.

## Consent

Written informed consent was obtained from the patient for publication of this case report and accompanying images. A copy of the written consent is available for review by the Editor-in-Chief of this journal.

## Competing interests

The authors declare that they have no competing interests.

## Authors' contributions

DS reported the initial clinical presentation and the diagnostic tests. CF reported the postoperative period and the follow-up. CF was the main contributor to this manuscript. MM conducted and described the surgical intervention. AB was responsible for, and reported on, the scintigraphies and commented on Figure 1. PY described histological findings, discussed pathological diagnosis, made available and commented on Figures 2, 3, 4, and 5. JP observed the patient’s progress, helped to report the initial presentation, reviewed the manuscript and contributed to the analysis of the literature. All authors read and approved the final manuscript.
